# A Refined Supervision Model of Rice Supply Chain Based on Multi-Blockchain

**DOI:** 10.3390/foods11182785

**Published:** 2022-09-09

**Authors:** Xiangzhen Peng, Xin Zhang, Xiaoyi Wang, Jiping Xu, Haisheng Li, Zhiyao Zhao, Zhibo Qi

**Affiliations:** 1 Beijing Key Laboratory of Big Data Technology for Food Safety, School of Artificial Intelligence, Beijing Technology and Business University, Beijing 100048, China; 2 Key Laboratory of Industrial Internet and Big Data, China National Light Industry, Beijing Technology and Business University, Beijing 100048, China; 3 Beijing Institute of Fashion Technology, Beijing 100048, China; 4 China Academy of Information and Communications Technology, Beijing 100048, China

**Keywords:** precision agriculture, rice supply chain, refined supervision, multi-blockchain, agricultural products, food safety

## Abstract

With the development of Agriculture 4.0, the requirements for sustainable agriculture and precision agriculture continue to grow. As one of the three major staple foods globally, the quality and safety of rice affect human health as well as social development. To ensure the quality and safety of rice and reduce the flow of problematic rice, a multi-layer blockchain-based rice supply chain refinement supervision model MBRRSM (Multi-blockchain Rice Refined Supervision Model) is proposed from the information level. First, the characteristics of information flow in the rice supply chain are analyzed, and a classification table of key information is constructed. Second, the MBRRSM framework is designed. Based on a multi-party hybrid encryption algorithm, secure multi-party computing algorithm, multi-mode storage mechanism, and SPOP (Supervision Proof of Peers) consensus algorithm, a set of mechanisms is designed for the transmission, use, storage, and consensus of rice data in MBRRSM. Subsequently, the security and performance capabilities of MBRRSM are analyzed. Meanwhile, the SPOP consensus algorithm is analyzed. Finally, a prototype system is built based on MBRRSM and verified through exemplary scenarios in different usage situations. The results show that research on the refined supervision of the rice supply chain based on multi-blockchain can finely supervise all types of data in the rice supply chain, and provide a guarantee for enterprise users to safely transmit and use data with different privacy levels. This study presents a unique research paradigm that introduces the theories and methods of the new information field generation into the field of agricultural research, and thus assists in the strategy implementation of “holding grain in the land and storing grain in technology”.

## 1. Introduction

Rice represents one of the world’s three major grains with global rice yields expected to reach 378,261-million kilograms by 2027, according to Statista. Sales of rice are expected to increase by 4.5% in 2023, and per capita consumption of rice in 2022 is expected to reach 42.4 kg. Consequently, considerations with regard to the food quality and safety of rice are therefore pertinent to human health [1,2,3]. In recent years, rice has been affected by heavy metals, mycotoxins, etc. and rice around the world is heavily polluted [4,5,6]. Starting from the circulation of information in the rice supply chain, the timely positioning, responsibility, and elimination of problematic rice can strengthen the supervision ability of various data relating to the rice supply chain and effectively reduce the quality and safety problems of rice products [7,8,9].

Agricultural scientific and technological innovation is one of the main measures to strengthen the more granular information in the field of agricultural products and food, which can promote the refined supervision of agricultural products and food, and ensure the quality and safety of agricultural products and food [10,11,12,13]. As a decentralized distributed storage ledger technology, blockchain uses the P2P network to broadcast information between nodes to achieve message synchronization of all nodes [14,15]. Multi-blockchain means that multiple blockchains are connected, and the synchronous transmission of messages in each blockchain node is realized through the cross-blockchain mechanism, multi-blockchain consensus mechanism, etc. [16,17]. Applying multi-blockchain to the information management of agricultural products and food supply chains can enhance the performance of blockchains, increase the accounting capabilities of blockchains, and realize the interconnection of multiple links in agricultural and food supply chains. Moreover, it can ensure the non-tampering and traceability protection of information in agricultural and food supply chains, and provide regulators with more accurate and comprehensive information supervision capabilities [18,19,20]. The research on the application of blockchain to agricultural products and food is mainly reflected in the following aspects. First, previous studies have explored the integrated application of blockchain and IoT technology, and explored the use of blockchain decentralization and IoT technology to jointly promote the digital transformation of agricultural and food supply chains [21,22,23]. Second, previous research has investigated the application of blockchain to the information management of different types of agricultural products and food, and provided information management and traceability services for different types of agricultural products and food supply chain structures [24,25,26,27]. Third, previous works have explored the integration and application of blockchain and neural network algorithms, big data technology, identification analysis technology, and other technologies to promote the fine management of agricultural products and food information [28,29,30,31]. Compared with the traditional centralized information supervision mode, the application of blockchain as a new scientific and technological innovation technology in the management of agricultural products and food supply chain can promote a more refined management of agricultural products and food, and provide feasible ideas for ensuring the quality and safety of agricultural products and food [32,33].

The rice supply chain is characterized by complex links, diverse participants, and redundant data. The application of blockchain has improved the ability of regulators to supervise rice supply chain data. Many scholars have conducted theoretical research, application research, architectural innovation, trusted interaction and efficient consensus research concerning blockchain implementation in the field of agricultural products and food. However, past research cannot be applied to the unique data flow characteristics of the rice supply chain. For example, references [34,35,36] illustrate the advantages of applying blockchain to the agricultural and food fields in theory, but ignore the many challenges that remain unresolved including performance and practical application. References [37,38,39] studied the application mode of blockchain in the field of agricultural products and food, but failed to address the complex supply chain characteristics of agricultural products and food. References [40,41,42,43] innovated and improved the blockchain architecture, but lacked details regarding the application, such as security, consensus efficiency, etc. References [44,45,46,47,48,49] improve the secure interaction of data and the efficiency of consensus, but the blockchain architecture they adopt renders it difficult to carry the complex data characteristics of agricultural and food supply chains. However, the increasingly frequent and diverse data requests and operations in the rice supply chain urgently need to improve the application of blockchain, mainly in the following aspects.

- For a sustainable rice supply chain, existing studies have been unsuccessful in their attempts to efficiently and securely supervise the complex links, data types, participants, and huge real-time data of the rice supply chain in a refined manner.
- The data suitability of the whole flow cycle of the rice supply chain is poor, and regulators are unable to accurately regulate different types of rice data in the rice supply chain by category. It is challenging to cover all types of data given the huge real-time data of the rice supply chain, and the granularity of the regulation of rice supply chain flow data is insufficient.
- The storage performance of blockchain is poor, and it is easy to cause data redundancy. In turn, this will lead to high latency and high concurrency problems, and the throughput of existing research cannot meet the frequent and complex data request requirements of the rice supply chain.
- The security and flexible application compatibility of rice supply chain data transmission based on blockchain is insufficient, and there are challenges in the secure classification, transmission, and application of basic rice data and private data. In addition, the demand for secure multi-party computing of various types of data between enterprises in all aspects of the rice supply chain has increased, and existing research cannot guarantee the safe use of private data for the relevant participating enterprises.

This paper first analyzes the rice supply chain and builds MBRRSM based on the “main chain + sub-chain” architecture in the multi-blockchain, which realizes the interconnection of all links and all types of data in the rice supply chain. Then, a data transmission mechanism is designed based on the multi-party hybrid algorithm to serve the secure interaction of various types of data in MBRRSM and the refined management of classification. Subsequently, based on the security multi-party computing algorithm, the data usage mechanism is designed to ensure that the participating companies in the rice supply chain can use it safely and multi-party without revealing their private data. Furthermore, a multi-mode storage mechanism is designed to serve the data classification storage of MBRRSM. Finally, the SPOP consensus mechanism serving the MBRRSM multi-chain architecture is designed to achieve complex and frequent consensus requests in the rice supply chain. This research coordinates the architectural innovation, application, data security interaction and efficient consensus of blockchain, which can be more applicable to the rice supply chain.

The rest of the paper is organized as follows. In Section 2, a literature review is conducted and analyzed. Section 3 presents the design of MBRRSM, including the analysis and framework of rice supply chain flow characteristics, data transmission mechanism, data usage mechanism, multimode storage mechanism, and the design of the SPOP consensus algorithm. Section 4 provides an analysis of the results, by analyzing the model as well as the prototype system, including the security, feasibility, performance, and the effect of the example application of MBRRSM, and demonstrates the role of MBRRSM in refined supervision of the rice supply chain.

## 2. Literature Review

With the development of blockchain technology, there are increasing numbers of research works on the application of blockchain in the field of agricultural products and food. The research on the application of agricultural products and food is mainly reflected in the theoretical research, architecture improvement, data application, data interaction, and consensus mechanism of blockchain in agricultural products and food supply chains, as shown in Table 1. Through the decentralization, traceability, tamper-proof, and other characteristics of the blockchain, the food quality and safety of agricultural products and food supply chains are guaranteed.

In terms of applied theoretical research in the field of agricultural products and food based on blockchain, the research focuses on the opportunities and challenges of blockchain research in the field of agricultural products and food. First, the application of blockchain in the field of agricultural products and food can enhance the sustainability of various agricultural products in three dimensions (i.e., economic, social, and environmental) [34]. Second, blockchain provides technological infrastructure such as digitization, automation, and tracking for agricultural and food supply chains. It can promote the digital transformation of agricultural products and food [35]. Finally, as a new generation of agricultural digital technology, blockchain can track and identify potential sources of pollution in the agricultural food supply chain [36]. The above research works have conducted quantitative and qualitative analyses through a large number of studies, and described the promotion effect of blockchain in the field of agricultural products and food. However, the supply chain of agricultural products and food is complex, and there are still many challenges in the real large-scale investment of blockchain in the field of agricultural products and food.

In previous works, the application research of blockchain in agricultural products and food has mainly focused on the use of blockchain to trace and manage agricultural product and food supply chain data. For example, Khan et al. proposed an optimized supply chain provenance system for Industry 4.0 in the food industry using state-of-the-art technologies such as IoT, blockchain, and advanced deep learning [37]. Zhang et al. designed a blockchain-based food supply chain security management system, pointing out that the use of blockchain is conducive to reducing management costs and improving management efficiency [38]. The above research pertains to the practical application of blockchain technology to agricultural and food supply chains. However, blockchain still faces many challenges concerning application, e.g., lack of government regulation and lack of trust among agricultural stakeholders in the use of blockchain, etc. [39]. Therefore, there is a need to improve existing blockchains to improve the applicability of blockchains and bring tangible benefits to traditional agricultural and food supply chains.

Therefore, in the previous studies, scholars have conducted research on the innovation and improvement of blockchain-based architecture in the agricultural and food fields. Research focuses on innovative design and application of new blockchain architectures to improve the scalability of blockchain in agricultural and food applications. For example, Leng et al. proposed a public chain of agricultural supply chain system based on double-chain architecture, and mainly studied the double-chain structure and its storage method, resource rent-seeking matching mechanism, and consensus mechanism [40]. Ali et al. proposed a sustainable blockchain framework for the halal food supply chain to overcome challenges related to blockchain implementation [41]. Song et al. proposed a two-blockchain structure including a consensus method, transaction mechanism, sustainability evaluation method, and performance optimization strategy [42]. The proposed structured was used to bring sustainable management practices of all players in to the blockchain network, especially allowing governments to play a more important role in agricultural supply management. Ren et al. proposed a dual-blockchain solution based on the interstellar file system storage for agricultural sampling data protection in IoT (Internet of Things) networks [43]. In summary, to cope with the complex supply chain structure of agricultural products and food, many scholars have begun to explore and improve the existing single-chain blockchain architecture. They use a multi-chain approach to solve the scalability problem of blockchain applications in the field of agricultural products and food, and some results have been achieved. However, the above research merely promotes the applicability of blockchain in the agricultural and food industry. The problem of data security interaction and coarse-grained enhancement of data supervision brought about by the double-chain structure has not been well solved.

In response to the above problems, scholars involved in previous studies have explored the research on trusted interaction of data in the field of agricultural products and food based on blockchain. The research mainly focuses on the integrated application of trusted encryption mechanism, Internet of Things technology and blockchain technology to ensure the trusted interaction of data. For example, Zeng et al. designed an efficient seed quality monitoring and smart water management system using IoT and blockchain technology to manage and coordinate the use of high-quality seeds and water resources in the community [44]. Vangala et al. designed a new authenticated key agreement mechanism envisioned in smart contract-based blockchains in the context of smart agriculture to enhance the persistence and auditability of data stored in blocks [45]. Durga et al. proposed a novel chaotic encryption-based blockchain IoT architecture to ensure data security and privacy in fields such as agriculture [46]. In the practical application of blockchain, the secure interaction of data is conducive to building trust among blockchain users, and the promotion and use of blockchain in the field of agricultural products and food.

Scholars have also conducted research on blockchain-based blockchain consensus in the field of agricultural products and food, which is used to improve the applicability of blockchain in the field of agricultural products and food. The research focuses on efficient consensus applicable to complex requests and participants in the agricultural and food sector. Xu et al. combined blockchain technology and wireless network technology to build a blockchain platform, combined with raft algorithm and PBFT algorithm to design an RBFT consensus mechanism to achieve “accurate identification” of poor people in agriculture [47]. Hu et al. used the immutability of blockchain and the paradigm of edge computing to build a trust framework for organic agricultural supply chains [48]. In addition, according to the organic agriculture supply chain scenario, all stakeholders are divided into four roles, and a new consensus mechanism is proposed to manage the information flow. Wang et al. proposed a practical Byzantine fault-tolerant consensus algorithm to score the credit of enterprise nodes in the rice supply chain, optimize the selection strategy of master nodes, and ensure high efficiency and low cost [49]. The blockchain consensus mechanism is one of the core mechanisms of the blockchain. Designing or improving the consensus mechanism to improve the performance of the blockchain is one of the means to improve the application efficiency of the blockchain in the field of agricultural products and food.

We divided the past research into five areas. Every aspect has contributed to the application of blockchain in agricultural products and food. However, the above research is narrow and cannot coordinate the practical application, architectural innovation, trusted interaction and efficient consensus of the blockchain. Therefore, it is impossible to better promote the digital transformation of blockchain services for agricultural products and food supply chains, and ensure the quality and safety of agricultural products and food. In this paper, we combine blockchain theory, new blockchain architecture, data encryption transmission, data security use and SPOP consensus mechanism to build MBRRSM. This is applied to the unique architecture of the rice supply chain, to serve the regulators to finely supervise the flow data of rice supply chain. Compared with other authors’ research on the application of blockchain to agricultural products and food, the main innovations of this paper are shown below.

- MBRRSM is built based on the main sub-chain architecture in the multi-layer blockchain to serve the refined supervision of the rice supply chain. Improvement in the auditability of rice supply chain data. Promotion of the precise control process of the rice industry. Sustainable development realization of the rice supply chain.
- The rice supply chain flow information is deconstructed and the key information is classified. The data transmission, usage, and storage mechanisms applicable to the rice supply chain are designed to improve the security of cross-chain data transmission and the granularity of regulation, satisfying the need for secure rice data computing and reducing data redundancy in the rice supply chain.
- The SPOP consensus mechanism is designed for MBRRSM based on the POP (Proof of Peers) consensus mechanism, which alleviates the frequent and complex data request consensus of each node in MBRRSM. It reduces the latency and concurrency of MBRRSM.

## 3. Model

### 3.1. Analysis of Rice Supply Chain

There are various types of rice, the circulation process is long and complicated, and there are many people involved. This paper investigates and analyzes the rice supply chain. First, the entire life cycle of rice is divided into nine links, including planting, acquisition, raw grain transportation, warehousing, processing, packaging, storage, transportation, and sales, according to the state of rice in different periods. Secondly, in the entire circulation cycle, the participants in the rice supply chain are mainly the participating companies, regulators, and consumers. This paper divides the participants in the whole life cycle of rice into the above three categories. Finally, this paper organizes the information circulating in the rice supply chain. According to the degree of privacy of the data generated by the rice supply chain, the data generated in the circulation cycle of the rice supply chain are classified, as shown in Table 2.

In this paper, the data are collected automatically and some of the data are entered manually using RFID (Radio Frequency Identification), sensors, thermometers, and other IoT devices, starting from the nine major segments of the rice supply chain and combining the participating companies, regulatory agencies, and consumers in each segment. The collected data are divided into two categories: basic data and private data. Private data include “co-use and co-ownership” data and “co-use and non-ownership“ data. This type of data can follow the flow of rice. The downstream enterprises of the same batch of rice can measure such data of the upstream enterprises, and thus cannot keep such data of the batch of rice completely confidential, which are “co-use” data. Moreover, this type of data is “co-ownership” to all enterprises involved in that batch of rice. Therefore, this type of data is “co-use and co-ownership“ data, and the data are generated from horizontal data collection in the rice supply chain, i.e., the same batch of rice is collected from different links. For “co-use and non-ownership” data, these include raw material costs, raw material sources, and chemical fertilizer names/sources. This type of data does not circulate with the flow of rice, and the affiliated company can encrypt this type of information, which are “non-ownership” data. However, this type of data has a “co-use” connection with all the enterprises involved in the batch of rice. Therefore, this type of data belongs to “co-use and non-ownership” data, and the data are generated from the vertical data collection of the rice supply chain, that is, different batches of rice, the same link data collection.

### 3.2. Framework

**Figure 1:**
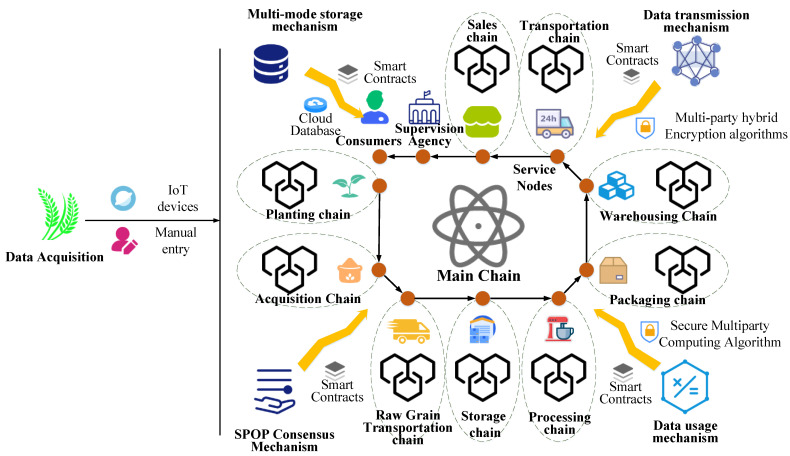
Schematic diagram of MBRRSM architecture.

The complex circulation process of the rice supply chain, the diverse participants, and the miscellaneous data types have brought certain difficulties to the refined supervision of rice supply chain information. Traditional blockchain research centers mostly on single-link blockchain applications, and it is difficult to supervise the entire circulation cycle of rice supply chain information. Due to the difficulty of data interaction between various links of rice, the refined supervision of all types of data in the rice supply chain by regulatory agencies will be affected. Therefore, based on the analysis of rice supply chain information, this paper builds MBRRSM based on Multi-blockchain. This model uses the Multi-blockchain architecture of “main chain + sub-chain”, which is suitable for the whole process flow of information in the rice supply chain. The design diagram of the MBRRSM framework is shown in Figure 1.

MBRRSM mainly serves the relevant rice supply chain regulators, enterprises, and consumers on the blockchain. Its framework includes “main chain + sub-chain” architecture, data transmission mechanism based on multi-party hybrid encryption algorithm, data usage mechanism based on secure multi-party computing algorithm, multi-mode storage mechanism based on “main chain + sub-chain + cloud database”, and consensus mechanism based on SPOP consensus algorithm. The model collects rice supply chain data automatically through IoT devices or through manual entry, and then standardizes the data through customized smart contracts. For the “main chain + sub-chain” architecture, the model consists of one main chain and nine sub-chains. The nodes of the main chain are divided into supervisory institution nodes, consumer nodes and service nodes of each sub-chain. The nine sub-chains include the planting chain, purchasing chain, raw grain transportation chain, warehousing chain, processing chain, packaging chain, storage chain, transportation chain and sales chain. These nine sub-chains run through the flow cycle of the rice supply chain, and the nodes in each sub-chain include enterprise nodes belonging to the type of the sub-chain and an information management node. Each sub-chain is connected to the main chain through a service node, which provides information refinement supervision services for the regulator. For the data transmission mechanism based on multi-party hybrid encryption algorithm, the multi-party hybrid encryption algorithm is designed to ensure the secure transmission between different data owners and different types of data in the “main chain + sub-chain” architecture. It is used to ensure the security of data transmission, and provides a way for supervisors to refine the data in real time. For the data usage mechanism based on a secure multi-party computation algorithm, this paper provides a set of secure multi-party computation mechanisms for “co-use and co-ownership” and “co-use and non-ownership” data, respectively, to ensure the secure usage of data. For the multi-mode storage mechanism based on “main chain + sub-chain + cloud database”, this paper provides a set of multi-mode storage mechanism for rice supply chain data triage. It increases the scalability of the model and reduces the latency of data interaction. For the SPOP consensus mechanism based on the SPOP consensus algorithm, this paper designs the SPOP consensus algorithm based on the POP consensus algorithm. It serves to reach consensus of nodes in MBRRSM, and is applicable to reach consensus of frequent and redundant data requests in the rice supply chain.

### 3.3. Data Transmission Mechanism

**Figure 2:**
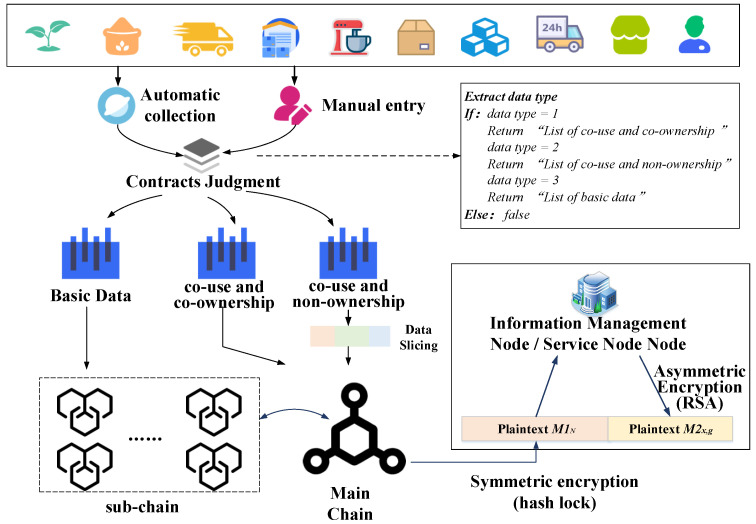
Schematic diagram of multi-party hybrid encryption.

The data type of the whole life cycle of the rice supply chain is divided into basic data and private data, among which the private data are divided into two types of data: “co-use and co-ownership” and “co-use and non-ownership”. To serve the secure and trustworthy data transmission between enterprises and the fine management of regulators, this paper designs a data transmission mechanism based on a multi-party hybrid encryption mechanism. The data transmission mechanism is divided into two steps: encryption and decryption, and the data encryption is shown in Figure 2.

Based on the unique data flow characteristics of the rice supply chain, this paper designs two interaction mechanisms for data encryption—hybrid encryption mechanism and multi-party hybrid encryption mechanism. Symmetric encryption algorithm and asymmetric encryption algorithm are used to encrypt the data, and the symmetric encryption algorithm adopts a hash-lock algorithm to facilitate data transmission across chains. The asymmetric encryption algorithm uses RSA (Public Key System) to reduce the amount of keys to be stored, reduce the difficulty of key distribution and meet the security requirements of private communication between enterprises that are unfamiliar with each other. The multi-party hybrid encryption mechanism serves the transmission of “co-use and co-ownership” data among various rice-related enterprises and the data storage in MBRRSM to ensure the secure storage and transmission of the “co-ownership” data in the “co-use” process. The encryption steps are shown below.

Hybrid encryption: It mainly serves the “co-use and non-ownership” data in private data and basic data. Encrypts the data transmission across the chain for individual enterprises. First, each enterprise initiates a request to store data on the blockchain, and the customized smart contract identifies the data collected automatically by IoT devices and the data types entered manually. In MBRRSM, “1” is “co-use and co-ownership” data, “2” is “co-use and non-ownership”, and “3” is basic data. Next, a random number *N* is generated and hashed to obtain *H (N)*. The custom-designed smart contract uses this hash lock to lock the data, generating plaintext *M*1*_N_*. Then, the sub-chain where this enterprise is located elects the information management node by consensus. This node is one of the enterprise nodes jointly elected by this sub-chain, and this information management node uses the RSA algorithm to encrypt the random number *N* for the first time to generate the plaintext *M*2*_x_*. The specific steps are: first select two unequal prime numbers *p* and *q*, and calculate *n* according to Equation (1). Calculate the Euler function $\varphi_{n}$ according to Equation (2). Select a random integer *m* such that 1 < *m* < $\varphi_{n}$, and with mutual prime. Calculate the modulo inverse element *j* of *m* for $\varphi_{n}$ according to Equation (3). Obtain the public key (*n*, *m*) and private key (*p*, *q*, *j*) of this information management node. Use this public key (*n*, *m*) to encrypt the random number *N*, perform the operation in Equation (4) from left to right for each digit *N_i_* of *N*. *k* is the maximum number of bits of *N* to get the plaintext *M*2*_x_*. *M*2*_x_* is used as the second encryption cipher for the second encryption.

(1) n=p×q

(2) $$\varphi_{n}=(p-1)(q-1)$$

(3) $$j=m^{-1}(\mathrm{mod}\varphi_{n})$$

(4) $$M2_{x}=\sum_{i}^{k}N_{i}\wedge m(\mathrm{mod}n)$$

Then, the service node corresponding to this sub-chain performs secondary encryption of *M*2*_x_* using the RSA algorithm. Since data interactions within the same sub-chain are carried out in parallel, one service node corresponds to multiple information management nodes. The information management node corresponding to each data request is required to sign the *M*2*_x_*. The service node uses the RSA algorithm to generate plaintext *M*2*_x,g_* for the signature certificate Signal and *M*2*_x_*. *M*2*_x,g,_* and *M*1*_N_* are arranged to form a mixed plaintext flow in MBRRSM. Applying this method of encryption ensures the cross-chain security of data. Secondly, the service nodes are managed by the supervisor, and this paper adopts the secondary encryption of information management nodes and service nodes, which reduces the centralization of the supervisor. All interaction information is managed by the supervision node, which increases the granularity of supervision by the regulators and realizes the refined supervision of rice supply chain data by the supervisors.

Multi-party hybrid encryption: It mainly serves privacy data, involving “co-use and co-ownership” data, and encrypts data of multi-party enterprises. After the data are automatically collected or manually entered, the data are sliced according to Equation (5), and the number of data slices *s_i_* generated is the number of parties that own the data.

(5) $$s_{i}=slice(\frac{long(Data)}{i})$$

Each *s_i_* is kept by a data owner, and each data slice is encrypted according to a hybrid encryption method. This approach combines both multi-party encryption and hybrid encryption, and each “co-use” of “co-ownership” data requires contractual consent from all data parties to restore and transmit the data. Again, the encryption is performed by the service node managed by the supervisor, adding granularity to the supervision of this type of data by the supervisor.

**Figure 3:**
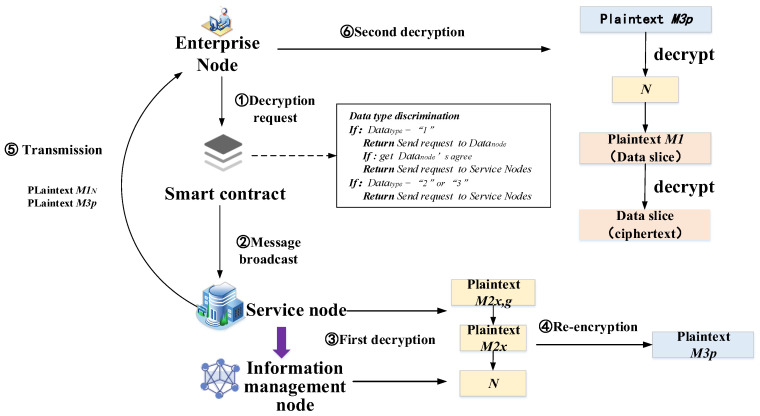
Schematic diagram of multi-party encryption mechanism decryption.

Using the hybrid encryption mechanism and multi-party hybrid encryption mechanism, the data are securely exchanged and stored in the “main chain + sub-chain + cloud database”. MBRRSM designs decryption steps for the above encryption methods, as shown in Figure 3.

The decryption process is divided into five main steps: decryption request, message broadcast, first decryption, re-encryption, transmission, and second decryption.

**Decryption request:** The enterprise node initiates a decryption request to MBRRSM.

**Message broadcast:** The contract determines the data type of the request, and for “co-use and co-ownership” data, the contract asks the data owner and broadcasts the request to the service node after receiving the consent message. For “co-use and non-ownership” data and basic data, the contract directly broadcasts the request to the service node.

**First decryption:** the service node decrypts with its private key and reduces each character segment in *M*2*_x,g_* according to Equation (6), *y* and *j* are a set of solutions in Equation (3). Separate *M*2*_x_* as well as the signed certificate.

(6) $$M2_{x}+Signal=\sum_{i}^{k}y_{i}\wedge j(\mathrm{mod}n)$$

Use *Signal* to locate the message management node. After verifying the security of the service node, this information management node decrypts *M*2*_x_* using the private key and obtains the ciphertext random number *N*.

**Re-encryption:** The service node uses the public key of the requesting enterprise node and re-encrypts *N* using the RSA algorithm to generate plaintext *M*3*_p_*.

**Transmission:** The service node performs cross-chain transmission to transfer *M*3*_p_*and *M*1*_N_* to the enterprise node of the data requester.

**Second decryption:** The data requestor first decrypts the plaintext *M*3*_p_* using the public key to obtain *N*. After that, it uses *N* to decrypt *M*1*_N_* to obtain the complete data or data slices.

The data interaction mechanism applied in MBRRSM is a multi-party hybrid encryption algorithm consisting of hybrid encryption and multi-party encryption fusion. This mechanism ensures the secure cross-chain transmission of data and adopts different encryption methods for different types of data. Under the condition that the supervisor ensures the refined management of the data transmitted in real time in MBRRSM, the centralization of MBRRSM is reduced by using the joint encryption of service nodes and information management nodes.

### 3.4. Data Usage Mechanism

The multi-party hybrid encryption algorithm-based data transmission mechanism ensures the trusted cross-chain transmission of basic data and private data in the rice supply chain and provides a refined management approach for supervisors. MBRRSM provides a secure multi-party computation mechanism when each enterprise participant in the rice supply chain needs to use certain private data of rice and does not want to leak their private data to other enterprises. This mechanism is implemented through Secure Multiparty Computation Contract-A (see Appendix A for contract pseudocode) and Secure Multiparty Computation Contract-B (see Appendix A for contract pseudocode). Secure Multiparty Computation Contract-A is for “co-use and non-ownership” data. Secure Multiparty Computation Contract-B is for “co-use and co-ownership” data and is a secure multiparty computation for horizontal data in the rice supply chain.

For “co-use and non-ownership” data, when the data are required for secure multi-party computation, the private data of the data owner are kept confidential. MBRRSM adopts a secure multi-party mechanism based on Shamir’s threshold secret sharing scheme to ensure secure multi-party computation of “co-use and non-ownership” data in the rice supply chain. First, the secure multi-party computation contract-A integrates the data of rice data owners according to Equation (7) to construct the multi-party computation of rice data *S*. *M_z_* is the private data of each rice data owner *P*. *z* is the number of enterprises participating in this multi-party computation.

(7) $$S=M_{1}+M_{2}+\ldots \ldots +M_{z}$$

The constant *t* is set as the minimum data owner’s consent required for data usage. Since MBRRSM uses the SPOP consensus mechanism with 51% fault tolerance, *t* is set to 1/2*z*. Construct the polynomial *F(x)* based on Equation (8), where *u* is prime and *S* is less than *u*. Take *z* unequal *x*’s and bring them into *F*(*x*) to obtain the *z*-group (*x_i_*, *y_i_*).

*F*(*x*) = S + a_1_*x^1^ + a_2_*x^2^ + …. + a_(t−1)_*x^(t−1)^mod(*u*) (8)

Each set of rice “co-use and non-ownership” data slices (*x_i_*, *y_i_*) is assigned to the data owner *P_i_* through the data transfer mechanism and *u* is made public. Finally, the polynomial *F(x)* is destroyed by the Secure Multiparty Computation Contract-A. When the data are used for computation, (*x_i_*, *y_i_*) greater than *t* group is brought into Equation (9) for rice data *S* reduction.

(9) $$F(x)=\sum_{i=1}^{t}(y_{i}\prod_{1\leq j\leq t,j\neq i}(x-x_{j})(\prod_{1\leq j\leq t,j\neq i}x_{j}-x_{i}){)}^{-1})\mathrm{mod}(u)$$

For “co-use and co-ownership” data, the data are shared by the data owners, so there is no need to perform confidential computation on the data. The secure multi-party computation of the data can be achieved by using the data transfer mechanism after the data owners reach a consensus. The request message containing the signature *R*_0_ of the requester is sent to the Secure Multiparty Computation Contract-B. The contract sends the request to each data owner node as *P_n_* based on the data transfer mechanism, and each data owner node sends the signature *R_n_* back to the Secure Multiparty Computation Contract-B. When the number of *R_n_* is reached, the data are used based on the data transfer mechanism.

MBRRSM ensures the secure computation of private data in the rice supply chain through the custom-designed Secure Multiparty Computation Contract-A and Secure Multiparty Computation Contract-B. It can guarantee the secure use of horizontal data as well as vertical data in the rice supply chain. This provides an idea to calculate and organize private data such as raw material cost, raw material source, and fertilizer name/source information in each link of the rice supply chain.

### 3.5. Data Storage Mechanism

**Figure 4:**
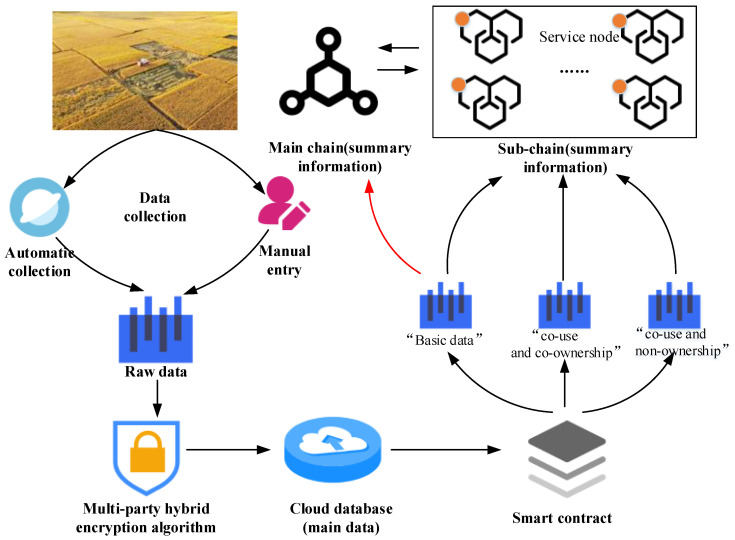
Schematic diagram of multimode storage mechanism.

MBRRSM, after data collection through IoT devices, stores and transmits data securely through the data transmission mechanism. The secure use of data is carried out through secure multi-party computing algorithms. In this paper, we design a multimode storage mechanism for MBRRSM and adopt the multimode storage mechanism of “main chain + sub-chain + cloud database”, as shown in Figure 4.

After the rice supply chain is automatically collected by IoT devices or entered manually, the data are first standardized and the raw data are obtained. The raw data are then encrypted using a multi-party hybrid encryption algorithm and stored in a cloud database, and a data summary *D_s_* is generated. Custom-designed smart contracts are used to classify the data, and the basic data as well as the private data are stored in a sub-chain. Considering that one enterprise may correspond to pairs of rice businesses in real applications, the same enterprise may have multiple enterprise nodes of sub-chains at the same time. MBRRSM custom-designed smart contract authenticates the enterprise and keeps one of the nodes as primary node and the rest as secondary nodes, and stores the data summary to the primary node. Each sub-chain has a service node, which is the channel for that sub-chain to link with the main chain. The privacy data are synchronized through the service node to the nodes that are not secondary nodes in other sub-chains. The basic data summaries are synchronized to the primary chain nodes, i.e., the supervisory nodes as well as the consumer nodes, through the service nodes. This approach achieves data streaming and ensures a sufficient number of data synchronization nodes. The service nodes are managed by the regulator, and these nodes are both main chain nodes and sub-chain nodes, and the regulator has all the data summary information after encryption. For consumers, MBRRSM sets the consumer nodes to access only the basic data, so it will not cause issues associated with data access error.

### 3.6. SPOP Consensus Mechanism

**Figure 5:**
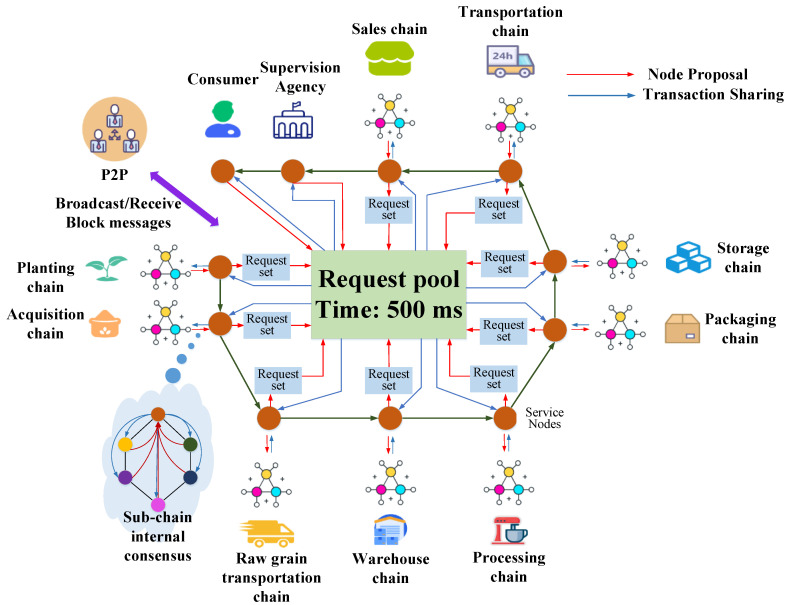
Schematic diagram of SPOP consensus algorithm.

All requests in MBRRSM need to reach a certain number of node consensus. In this paper, we improve the POP (Proof of Peers) consensus mechanism of chainsql application and design the SPOP consensus algorithm for MBRRSM. It is more suitable for MBRRSM and for the complex and frequent rice supply chain data interaction requests in MBRRSM, and the SPOP consensus algorithm is shown in Figure 5.

The SPOP consensus algorithm process consists of six steps, as follows.

(1) Each node collects requests: for the supervisor node and the consumer node, the nodes on the main chain: propose their requests to the request pool. For each sub-chain internal node: each node submits its request to the request set, and the service node proposes the sub-chain request set to the request pool.
(2) According to different node data permissions, each supervisor node, consumer node, and service node continuously extracts requests from the request pool. Each supervisory node, consumer node, and service node looks at the request pool header information, determines which requests are not available in the request set within its authority, and extracts the requests that are not available.
(3) Each service node broadcasts requests to the corresponding sub-chain network. Each enterprise node looks at the request set header information, determines the requests that are not in its own request set, and extracts the requests that are absent.
(4) Each enterprise node votes on the request and broadcasts its vote
(5) Each node collects votes from other nodes and when it exceeds 51%, each node reaches consensus and the request is executed.
(6) Each node generates blocks based on the request pool and broadcasts the new blocks

To meet the processing efficiency of MBRRSM for data requests in the rice supply chain, the fault tolerance mechanism of the SPOP consensus algorithm designed in this paper is set as follows: the initial trial time is set to 0, and each Time determines whether the request pool consensus times out, and the timeout time is set to 500 ms. The timeout duration is configurable, and combined with the demand of MBRRSM test requests, the initial trial is set to 500 ms in this paper. Within the specified time, when the request fails to reach 51% of the votes, the request will return to the request pool to wait for the next consensus reconfirmation, and the consensus is reached when the request successfully obtains 51% of the votes.

## 4. Results

### 4.1. Model Analysis

#### 4.1.1. Security Analysis

MBRRSM adopts a multi-chain architecture and designs a data transfer mechanism, a data usage mechanism, and a multi-mode storage mechanism. The SPOP consensus algorithm is custom selected and improved to jointly serve the secure interaction of data in MBRRSM. It provides a real-time and secure refined management approach for supervisors to monitor data. We tested and verified the security performance of MBRRSM. First, 30,000 data requests in a certain time period were selected for attack-resistance testing. The Sybil attacks, DDOS (Distributed Denial of Service) attack and smart contract attack were tested, respectively. Sybil attacks include creating fake nodes and publishing fake data. DDOS attacks attempt to modify the blockchain client or server software, passively wait for decryption requests from other nodes, and then attack by returning false responses. Smart contract attacks are set up to attempt to modify smart contract-related parameters. We recorded the responses of the system, as shown in Table 3.

In the 30,000 sets of test data, MBRRSM was able to achieve timely identification of false nodes as well as false data by custom-designed multi-party hybrid encryption mechanism, and supervisors were able to completely resist 200 Sybil attacks by monitoring service nodes in real-time and supervising abnormal data in real-time. Through the multi-party hybrid encryption mechanism, the decryption of data needs to pass the smart contract review, service node review, and information management node confirmation in turn. In addition, the decryption process employs the data user’s private key for secondary encryption transmission. Finally, decryption is performed through the hash lock. The decryption complexity of data is extremely high. Among 200 DDOS attacks, the number of times the data are successfully attacked is 0. For smart contract-related parameter modification attacks, the smart contract parameter modification allowed by MBRRSM only involves consensus batch processing time. This will only have an impact on the performance and will not have any impact on the security of the data. The number of nodes in MBRRSM is so large that the possibility of successful smart contract attacks is close to 0. In 200 attack tests, the success rate is 0%.

#### 4.1.2. Analysis of Model Runs

**Figure 6:**
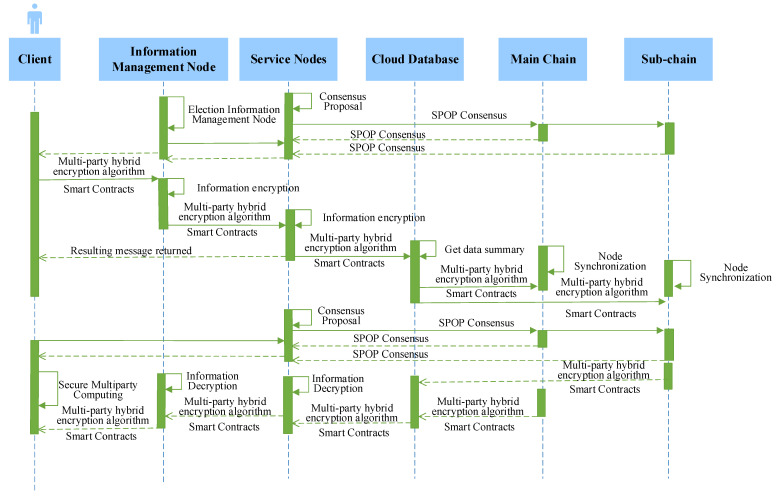
MBRRSM Logic Timing Diagram.

MBRRSM is designed as a “main chain + sub-chain” architecture, which is suitable for a complex rice supply chain with a variety of participants and a large amount of real-time data. This section analyzes the operation flow of MBRRSM. Figure 6 shows the logical timing diagram of MBRRSM, which shows that MBRRSM is a fully feasible solution.

For data collection, first, the user initiates a request from the client. After the smart contract verifies the user’s authority as well as the request type, the data transmission mechanism based on multi-party hybrid encryption algorithm is used to encrypt the transmission. Each enterprise within the same sub-chain elects the information management node of that sub-chain regarding this request by initiating SPOP consensus. Together with the service node, the request information is encrypted, and the supervisor refines the management of this request through the service node. Secondly, data requests for different data types are synchronized to the corresponding blockchain through customized smart contracts. For the use of data, the user’s request is either securely multi-party computed from the user side after the SPOP consensus, or MBRRSM feeds the encrypted data digest as well as the plaintext data. The data are interacted using the decryption step of the multi-party mixed encryption algorithm.

MBRRSM adopts the architecture of “main chain + sub-chain” and divides the sub-chains according to the service types of rice supply chain enterprises, which enhances the linkages of rice business between enterprises. The model complexity is O(N^4^), which ensures the security of data interaction in the model. When faced with scenarios where multiple sub-chains may exist in one enterprise in the actual application process, MBRRSM customized smart contract provides for all nodes of the same enterprise and only retains one master node as the data synchronization node, reducing data redundancy. The data interaction adopts multi-party hybrid encryption to ensure the secure interaction of different types of data. Based on the encryption of data owner nodes, information management nodes, service nodes and supervisor nodes are added to jointly encrypt data, which reduces the centralization of the model. For the refined management of the supervisor, all requests are directly contacted by the supervisory nodes through the service nodes of each sub-chain, and the supervisor’s ability to control the real-time data of the rice supply chain is enhanced. Finally, MBRRSM supports consumer uplink, which increases the transparency of rice data and enhances the credibility of supervisors as well as enterprises.

#### 4.1.3. Information Consensus Analysis

The SPOP algorithm is designed based on POP algorithm, thus allowing it to be applied to MBRRSM operation to guarantee the consensus of frequent and complex data interaction requests in the rice supply chain. The POP consensus algorithm is the RPCA (Ripple Consensus Algorithm) consensus algorithm improved by chainsql using PBFT consensus algorithm. In this paper, we use a common 8-core 16 G environment, to test the consensus performance of SPOP, where the number of nodes is set to 4 and MBRRSM arranges 1 child chain and 1 main chain for testing. It is compared with the POP consensus algorithm and RPCA algorithm, as shown in Table 4.

The SPOP algorithm designed in this paper demonstrates a significant improvement in sending tps, consensus tps and incoming tps compared with RPCA consensus algorithm and POP consensus algorithm. Moreover, the SPOP consensus algorithm increases the request pool and sub-chain request set to increase the processing capability of the model for requests, which is more suitable for the frequent data interaction requests in the rice supply chain. Using the mixed proposal mechanism of main chain nodes (supervisory nodes, consumer nodes, and service nodes) is more suitable for the special architectural characteristics of the rice supply chain. In addition, SPOP consensus algorithm verifies all kinds of consensus requests in MBRRSM only once, and its algorithm complexity is O(N), thus shortening the block-out time of MBRRSM.

**Figure 7:**
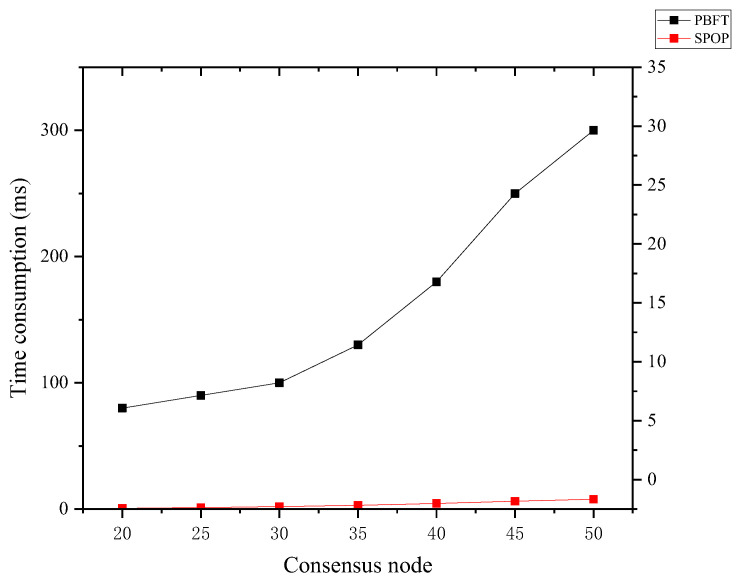
Comparison of consensus time consumption of SPOP consensus algorithm.

To test the practical application capability of SPOP, we arranged nine corresponding main chains as well as a sub-chain. With an increase in the number of nodes, the change in consensus consumption time is shown in Figure 7. We compare the SPOP consensus algorithm with the traditional PBFT consensus algorithm, which is used to show the advantages of the SPOP consensus algorithm more intuitively. This test MBRRSM involves 9 sub-chains and 1 main chain, so the number of SPOP consensus nodes is arranged as 20, including 9 service nodes, 1 regulator node, 1 consumer node, and 1 enterprise node in each sub-chain. The PBFT consensus mechanism is also tested from 20 nodes.

The SPOP consensus algorithm demonstrates excellent performance and is suitable for MBRRSM’s multi-chain architecture to meet the demand volume of rice supply chain interaction requests. From Figure 7, we can see that when the consensus nodes reach 50, the consensus time consumption of PBFT consensus algorithm has reached 300 ms. The consensus consumption of SPOP consensus algorithm is only about 2 ms and smoother. PBFT belongs to the way of leader proposal, SPOP is more decentralized compared to PBFT class consensus algorithm.

In terms of fault tolerance, we compare SPOP consensus algorithm with PBFT consensus algorithm, RPCA consensus algorithm, and POP consensus algorithm as shown in Table 5.

PBFT consensus algorithm and RPCA consensus algorithm use two rounds of 2/3 consensus with 33% fault tolerance. POP consensus algorithm uses one round of consensus, each node proposes to each node, only the number of nodes that reach consensus exceeds 51%, and then consensus can be reached. SPOP consensus algorithm is used in the multi-chain architecture of MBRRSM, which adopts a mixed proposal mechanism of main chain nodes (supervisor node, consumer node, and service node), that is, each node in the sub-chain proposes to the service node, and the service node proposes the request set to the request pool, and its consensus tolerance is also 51%. In addition, the consensus requires more than half of the nodes to reach the same consensus.

#### 4.1.4. Refined Supervision Analysis

**Figure 8:**
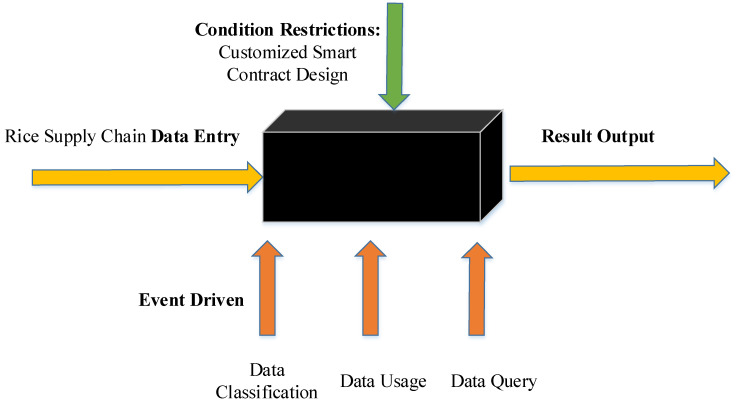
Schematic diagram of black-box testing.

To analyze the practical application effect of the model, this study uses black-box testing to verify the ability of accurate classification, safe transmission and safe use of three types of data. The schematic diagram of black-box testing is shown in Figure 8.

Using black-box testing, we analyzed the model designed in this study. The analysis results are shown in Table 6.

Through black-box testing, the model designed in this study is tested for accurate data classification, secure transmission and usage. The tested cases all achieved the expected goals. Compared with the traditional blockchain supervision model, the model realizes the safe transmission, use and storage of data, and achieves the precise supervision of data.

### 4.2. Prototype System Design and Verification

#### 4.2.1. Prototype System Construction

This paper builds the MBRRSM prototype system based on MBRRSM. The Linux version is 16.0.0 and the Ubuntu version is 20.04.1. On this basis, Fabric 2.1 is used to build. The Docker version is 20.10.7, the docker-compose version is 1.25.0, the Go language version is 1.17.2, and the storage medium is cloud database. The MBRRSM prototype system uses React.js, Vue.js, and AngularJS to develop the front-end of the system. MBRRSM contains a total of one main chain and nine sub-chains. The prototype system is designed as a five-layer architecture, including data acquisition layer, network layer, consensus layer, smart contract layer and application layer. The data collection layer is the data collection method and related IoT devices, the network layer is the P2P transmission method, the consensus layer is the SPOP consensus mechanism, and the smart contract layer encapsulates the secure multi-party computing smart contract and so on. For the application layer, the user can log in by selecting the appropriate identity and by entering the account password and other information. In addition, users can sort and upload rice data according to three data types: basic data, “co-use and co-ownership” data, and “co-use and non-ownership” data. The system can correct the input data format in real time. In terms of data interaction, after receiving the request, the user can view the data to be decrypted, enter their private key to decrypt the data, and the decryption progress is displayed through the visualization progress ring.

#### 4.2.2. Business Scenario Application Analysis

MBRRSM can provide services for the interaction of basic rice supply chain data, “co-use and co-ownership” data, and “co-use and non-ownership” data. After a field inspection of a batch of rice in northeast China, the batch of rice has a complete data record. Through this batch of rice data records, the example test of the MBRRSM prototype system is mainly divided into three business scenarios for testing, including the test of basic data, the test of “co-use and co-ownership” data, and the test of “co-use and non-ownership” data.

**Business Scenario I**

**Figure 9:**
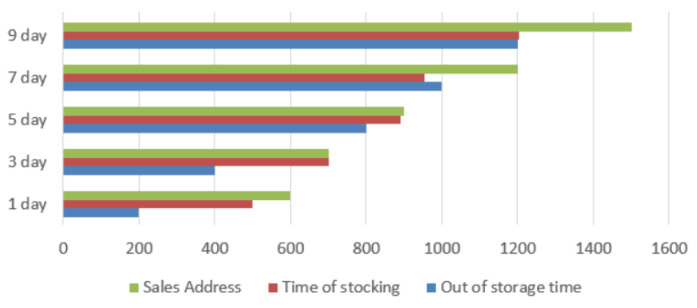
Statistics of the number of queries.

Business scenario I corresponds to the basic data in the rice supply chain, including application tests on basic data such as the time of rice release, logistics departure time, arrival time, sales address, and purchase time. The tests include consumer traceability inquiries, as well as fine-grained supervision tests by regulators. We counted the number of traceability queries for each sub-category of data for the batch of rice for each day over a 10-day period, as shown in Figure 9. The specific query situation of each category of data can be visualized. As the system lengthens, the amount of data traceability increases every day. From the back-end of the system, the supervisory agency node can pay attention to the time and content of each query in real-time, and can achieve refinement of the basic information management.

**Business Scenario II**

**Figure 10:**
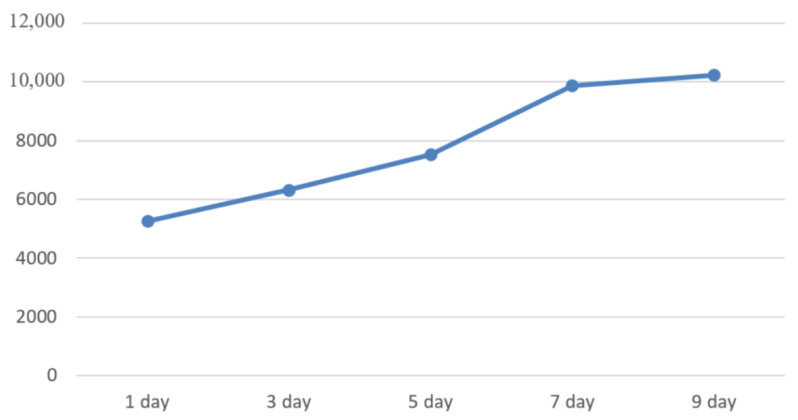
Statistical chart of trend changes in data interaction volume.

Business scenario II corresponds to the “co-use and co-ownership” data in the rice supply chain, including the application test of horizontal data of the rice supply chain such as hazard information and rice quality information. The trend of the number of interactions of these data in the system over the 10-day period is shown in Figure 10. It can be seen that the application of this system has contributed to the interconnection and mutual use of data in the rice supply chain, resolving the problem of information silos in the traditional model. The amount of data interaction within the system is greater in comparison to the single-layer blockchain architecture, which likewise reflects the lower processing latency of this system for various types of requests. For the supervisor, it can monitor and manage each of the data in the system in a more refined way, and can locate and deal with abnormal data promptly.

**Business Scenario III**

Business scenario III corresponds to the “co-use and non-ownership” data in the rice supply chain, including the application testing of raw material cost, raw material source, fertilizer name/source, planting/harvesting time, light level, and watering records, etc., for each link. In this paper, we test the multi-party secure computing capability of each company in MBRRSM for this type of longitudinal data for testing. We calculate the average value of the data related to 6 different batches of rice owned by 12 participating companies in the growing segment of the rice supply chain. The MBRRSM was able to securely obtain the calculation results without disclosing the information of each participating company, as shown in Table 7.

### 4.3. Discussion

There have been many studies on applying multi-chain architecture to increase the scalability of blockchain in agricultural products and food. The agricultural supply chain system based on a dual-chain architecture proposed in Ref. [36] can balance the openness and security of transaction information and the privacy of enterprise information, adaptively complete the rent-seeking and matching of resources, and improve the credibility of the public service platform and overall efficiency of the system. Tao et al. proposed a food safety supervision system with a hierarchical multi-domain blockchain network structure and a secondary inspection mechanism, which is capable of automatic food quality detection and early warning of unqualified food products in the whole industry chain [50]. We present a comparative analysis of the MBRRSM designed in this study as described in Table 8.

In Ref. [36], a public blockchain of agricultural business resources based on “user information chain” and “transaction chain” was designed to achieve information streaming. This reduces the redundancy of data and increases the throughput of the system to a certain extent. However, the bifurcation of information causes lower attack diversity and attack cost compared to full node backup. Based on the POS consensus algorithm, the authors propose a consensus algorithm that considers the weight, which reduces the resource consumption to a certain extent, but its application scalability is low. Tao et al. designed a two-level verification mechanism as well as a supervisory node election and replacement mechanism based on the PBFT consensus mechanism with a low fault tolerance of 33% [50]. The authors used a two-level verification mechanism as well as a sub-master chain architecture to make its block propagation complexity O(m^2^ + x^2^), which improves the attack diversity as well as the attack cost of the food safety supervision system. However, this also resulted in high resource consumption. In addition, because of the PBFT consensus mechanism, the throughput remained low despite the improvements made. In terms of scalability, the architecture studied by the authors is generic, so its application scalability is high. Additionally, in this study, in terms of security, MBRRSM applies the SPOP consensus mechanism, which bears a higher fault tolerance rate of 51%. Multi-party hybrid encryption, secure multi-party computation, and smart contracts are used, the complexity of encrypted transmission is O(N^4^), and both the attack diversity and attack cost is higher, thus the data security is higher. In terms of model efficiency, this model uses SPOP consensus algorithm and the model throughput can reach tps, which is higher compared to the throughput reported in Refs. [36,50]. In terms of scalability, MBRRSM consumes more resources, but adopts methods such as increasing transaction sets and transaction pools, which improved resource consumption. For application scaling, the three mechanisms involved in MBRRSM and the SPOP consensus algorithm can provide a feasible idea for secure data transmission and application, as well as refined management aspects for the whole supply chain industry.

## 5. Conclusions and Future Work

The application of multi-blockchain architecture to the fine-grained supervision of rice supply chain information presents a unique research paradigm in the new generation of information technology applied to the field of agricultural information management. It is a unique research concept for the application of next-generation information technology in the field of agricultural information management. It audits and supervises rice data at the information level, thus ensuring data security. The MBRRSM is used for data collection, data transmission, data usage and data storage in the rice supply chain. The data transmission mechanism based on multi-party hybrid encryption algorithm facilitates the classification and transmission of different types of data and increases the supervision of rice data by the regulatory structure; the data usage mechanism based on secure multi-party computing algorithm facilitates the credible use of different types of private data among enterprise users; the multi-mode storage mechanism based on “main chain + sub-chain + cloud database” facilitates the secure storage of redundant data in the rice supply chain; the consensus mechanism based on the SPOP consensus algorithm facilitates the storage of redundant data. MBRRSM provides a feasible idea for the precise regulation and sustainable development of agricultural and food supply chains.

In terms of economic feasibility, MBRRSM adopts a multi-blockchain architecture of “main chain + sub-chain”. Compared with the single-layer blockchain architecture, MBRRSM’s multi-layer blockchain architecture has lower maintenance cost, and only one maintenance mechanism is needed to maintain the entire flow chain, participants and flow data of the rice supply chain. In addition, MBRRSM adopts the multi-mode storage mechanism of “main chain + sub-chain + cloud database”, which is more intelligent than the traditional centralized storage mechanism, requires lower labor cost, and is safer and more efficient.

Although the proposed MBRRSM can allow regulators to refine the regulation of rice supply chain information, the model still bears some limitations.

- MBRRSM can guarantee the security of the data automatically collected by the rice supply chain before storing it on the blockchain. However, for some manually entered information, maintaining the security before storing in the blockchain can only be achieved by standardizing the data and training employees within the company. In the future, achieving credible storage of data on top of the blockchain will be a direction of research.
- MBRRSM is a unique supply chain architecture for the complexities of the rice supply chain, the many people involved, and the many types of data. Some modifications to MBRRSM are needed when extending it to other food crops as well as to agricultural and food supply chain regulations.
- MBRRSM supports consumer on-chain and can provide commodity traceability services for consumers, etc. However, multi-layer blockchain is an innovative application of new-generation information technology in the field of agricultural products and food. Ways to improve consumer trust of this new technology remains an important future consideration.

In the future, after proper modification targeting the limitations of MBRRSM, it can be combined with cutting-edge technologies in other fields such as hyperspectral rapid detection technology, Internet of Things technology, identification resolution technology, and digital twin technology. In doing so, this research can better serve the supervision of food quality and safety in the field of agricultural products and food. This research will contribute to the development of Agriculture 4.0 at the digital level.

## Figures and Tables

**Table 1:** Literature review table.

Category	Main Content	References
Blockchain-based theoretical study of applications in the field of agricultural products and food	Analyze the existing research and study the opportunities and challenges of blockchain research in the field of agricultural products and food.	[34,35,36]
Blockchain-based data application methods in the field of agricultural products and food	Focuses on the use of blockchain to trace and manage agricultural products and food supply chain data.	[37,38,39]
Blockchain-based architecture innovation and improvement research in the field of agricultural products and food	Innovative design and application of new blockchain architecture to improve the scalability of blockchain in agricultural and food applications.	[40,41,42,43]
Blockchain-based research on the trusted interaction of data in agricultural products and food	Research the integrated application of trusted encryption mechanism, Internet of Things technology, etc., with blockchain technology to ensure the trusted interaction of data.	[44,45,46]
Blockchain-based consensus research in the field of agricultural products and food	Research applies to complex requests and efficient consensus among participants in the field of agricultural products and food.	[47,48,49]

**Table 2:** Data analysis table of rice circulation cycle.

**Basic Data**		Product name/lot, inbound time, worker cost, outbound time, logistics departure time, arrival time, sales address, inbound time, selling time, product quantity, etc.
**Privacy data**	**co-use and co-ownership**	Hazard information (mycotoxins, heavy metals, pesticide residues, fumigant residues, herbicide residues, molds), rice quality information (drought resistance, flood resistance, yield, pollination rate, cold resistance), whole rice/broken rice rate, etc.
	**co-use and non-ownership**	Raw material cost, raw material source, fertilizer name/source, planting/harvesting time, light level, watering record, purchase inspection report, drying record report, temperature and humidity record, processing cost, rice milling/color selection/polishing method, packaging cost, transportation cost, transportation vehicle information, route information, driver information, storage cost, herbicide dosage, fumigant dosage, etc., for each link

**Table 3:** Attack test table.

Attack Method	Sybil Attack	DDOS Attack	Smart-Contract Attack
**Number of attacks**	200	200	200
**Number of successes**	0	0	0
**Percentage of successful attacks**	0%	0%	0%

**Table 4:** SPOP Consensus Performance Test Table.

Performance	Sending tps	Consensus tps	Inbound tps
RPCA consensus algorithm	700	700	500
POP consensus algorithm	4000	4000	1800
SPOP consensus algorithm	6000	6000	2900

**Table 5:** Comparison table of algorithms.

Category	PBFT Consensus Algorithm	RPCA Consensus Algorithm	POP Consensus Algorithm	SPOP Consensus Algorithm
**Consensus method**	Two rounds of 2/3 consensus	Two rounds of 2/3 consensus	Individual proposals for each node	Hybrid proposal mechanism for master chain nodes (supervisory nodes, consumer nodes, service nodes)
**Fault** **tolerance**	33%	33%	51%	51%

**Table 6:** Black-Box Test Results Table.

Test Target	Case Number	Feature Points	Test Content	Expected Outcome	Test Results
Data classification	Case1	Precise classification of data	Accurate classification by contract after standardization of collected data	The data are accurately classified into the category according to the category.	As expected
Data transmission	Case2	Secure data transmission	Safe transmission of private data without leakage	Data are transmitted securely, without tampering or leakage.	As expected
Data usage	Case3	Safe use of data	Data can be securely used for computation among multiple stakeholders	The data are used as expected.	As expected

**Table 7:** Table of calculation results of safety multiple.

Category	Seed Cost	Illumination	Watering Frequency	Fertilizer Cost
**Average value**	RMB 5.25/kg	46,500 lux	1.25 times/week	RMB 345/ton

**Table 8:** Comparative analysis table.

Performance	Indicators	Ref. [36]	Ref. [50]	Our Study
**Security**	Fault tolerance	middle	low	high
	Attack Diversity	low	high	high
	Attack Cost	low	high	high
**Model efficiency**	Throughput	high	low	high
**Scalability**	Resource consumption	middle	high	middle
	Application Scalability	low	high	high

## Data Availability

The data supporting the findings of this study are available upon request from the authors.

## Appendix A

### Appendix A.1. Algorithm A1

**Algorithm A1** Secure Multiparty Computation Contract-A
**1: Data Reconfiguration** **If** receive (*M_z_*) //Accept data per data owner *S* = *M*_1_ + *M*_2_ + *M*_3_ + ……//Build multi-party computing data *S* **Return** “Successful data construction” **Else** “Data build failure” **2: Generate a new *S*-slice** *t* = 1/2*z*//MBRRSM multi-party fault tolerance is set to 51%, which means that at least 1/2 of the nodes of the data owner are required. *F*(*x*) = S + a_1_*x^1^ + a_2_*x^2^ + …. + a_(t−1)_*x^(t−1)^mod(*u*)//Constructing polynomials *F*(*x*) **If** Take *z* unequal *x*’s and bring them into *F (x)* **Return** *z* (*x_i_, y_i_*) **If** Send (*x_i_, y_i_*) ->*P_z_*//Assigned to the data owner *P* Public *u* **Return** Destruction of *F(x)* **3: Data Sharing** **If** *x* = 0 **Return** *S* **If** receive (*x_i_*, *y_i_*) (*x_i_, y_i_*) >=*t* **Return** send (*x_i_*, *y_i_*) ->*F(x)//*Restore shared non-common data *S* $F(x)=\sum_{i=1}^{t}(y_{i}\prod_{1\leq j\leq t,j\neq i}(x-x_{j})(\prod_{1\leq j\leq t,j\neq i}x_{j}-x_{i}){)}^{-1})\mathrm{mod}(u)$ **Return** *S*

### Appendix A.2. Algorithm A2

**Algorithm A2** Secure Multiparty Computing Contract-B
**1: if** Data usage request received Query the number of members the secret has *z* **For** (i = 0, i < =z, i++) { i = 0; Send(request)_R0_->P_z_; i++; }//Send request messages to each data owner node **Return** “Sending successfully” **Else** “Sending failure” **2: if** Receive (Sig (Request) Rz) //Feedback from Data Owners **If** Sig (Request) _Rz_ = z **Return** “Allow requests” **Else** “Request failed” **Else** “No feedback”
